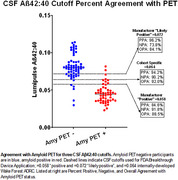# Approaches to a center‐specific CSF amyloid ratio cutoff based on PET‐amyloid positivity at the Wake Forest Alzheimer's Disease Research Center

**DOI:** 10.1002/alz70856_107322

**Published:** 2026-01-09

**Authors:** Courtney L. Sutphen, Marc D. Rudolph, Melissa M. Rundle, Christopher T Whitlow, Kiran K. Solingapuram Sai, Timothy M. Hughes, Trey Bateman, Sam N. Lockhart, Xiaoyan Iris Leng, Suzanne Craft, Michelle M Mielke, Thomas C. Register

**Affiliations:** ^1^ Wake Forest Alzheimer's Disease Research Center, Winston‐Salem, NC, USA; ^2^ Wake Forest University School of Medicine, Winston‐Salem, NC, USA; ^3^ Perceptive Inc., Burlington, MA, USA; ^4^ Division of Public Health Sciences, Wake Forest University, School of Medicine, Winston‐Salem, NC, USA

## Abstract

**Background:**

Variation in demographics and regional influences on AD suggest a need for ADRC center‐specific biomarker cutoffs. The Wake Forest ADRC has a unique community‐based cohort from a recruitment area with high rates of metabolic disease and hypertension. We evaluated the accuracy and utility of the CSF Aβ42:Aβ40 ratio (Lumipulse G1200 platform) to classify participant amyloid positivity using (1) general cutoffs developed for the assay manufacturer for a Breakthrough Device application to the FDA (≤0.058 “positive” and ≤0.072 “likely positive”), and (2) internal amyloid PET visual reads. The FDA cutoffs were developed using 235 Amsterdam Dementia Cohort participants and cross‐validated in 274 cognitively impaired ADNI participants. We evaluated performance of a center‐specific CSF Aβ42:40 cutoff in our unique cohort, which includes cognitively impaired and unimpaired participants, compared to generalized cutoffs.

**Method:**

113 participants (enrolled 2014‐2023) had baseline lumbar puncture and amyloid PET. Amyloid PET positivity was adjudicated based on visual read agreement between two expert independent readers; *n* = 61 participants adjudicated as PET‐positive were significantly older and had greater APOEε4 carriage, but no difference in education or sex. First‐thawed aliquots of CSF were assayed for Aβ1‐40 and Aβ1‐42 and the ratio of Aβ42:Aβ40 was calculated. An internally validated cutoff was developed using ROC analysis to calculate Youden's J‐index, giving the amyloid ratio cutoff that best aligned with amyloid PET. The percent positive, negative, and overall agreement (PPA, NPA, OPA) was calculated for each cutoff. The cutoff reported here is not adjusted for age or APOEε4.

**Result:**

For the FDA application “positive” and “likely positive” cutoffs, respectively, PPA with amyloid PET was 84.6 and 96.2%, NPA was 91.8 and 73.8%, and OPA was 88.5 and 84.1%. The center‐specific cutoff was ≤0.064, resulting in PPA of 94.2%, NPA of 90.2%, and OPA of 92.0%.

**Conclusion:**

It is feasible for the Wake Forest ADRC to adopt a center‐specific CSF amyloid ratio cutoff for use in our research studies. The proposed cutoff of ≤0.064 will be used in a larger dataset of all participants with baseline CSF (*N* = 178) to estimate appropriate center‐specific cutoffs for CSF total Tau and pTau181, and plasma amyloid ratio and pTau217.